# CCDC157 is essential for sperm differentiation and shows oligoasthenoteratozoospermia‐related mutations in men

**DOI:** 10.1111/jcmm.18215

**Published:** 2024-03-20

**Authors:** Huimei Zheng, Chenjia Gong, Jingping Li, Jiaru Hou, Xinhan Gong, Xinhai Zhu, Huan Deng, Haoyue Wu, Fengbin Zhang, Qinghua Shi, Jianteng Zhou, Baolu Shi, Xiaohang Yang, Yongmei Xi

**Affiliations:** ^1^ Division of Human Reproduction and Developmental Genetics, the Women's Hospital Zhejiang University School of Medicine Hangzhou China; ^2^ Hefei National Laboratory for Physical Sciences at Microscale, the First Affiliated Hospital of USTC, USTC‐SJH Joint Center for Human Reproduction and Genetics, The CAS Key Laboratory of Innate Immunity and Chronic Diseases, School of Life Sciences, CAS Center for Excellence in Molecular Cell Science, Collaborative Innovation Center of Genetics and Development University of Science and Technology of China Hefei China; ^3^ Institute of Genetics Zhejiang University Yiwu China; ^4^ Center for Genetic Medicine, the Fourth Affiliated Hospital Zhejiang University School of Medicine Yiwu China; ^5^ College of Life Sciences Zhejiang University Hangzhou China

**Keywords:** acrosome, CCDC157, Golgi apparatus, oligoasthenoteratozoospermia, spermatogenesis

## Abstract

Oligoasthenoteratospermia (OAT), characterized by abnormally low sperm count, poor sperm motility, and abnormally high number of deformed spermatozoa, is an important cause of male infertility. Its genetic basis in many affected individuals remains unknown. Here, we found that *CCDC157* variants are associated with OAT. In two cohorts, a 21‐bp (g.30768132_30768152del21) and/or 24‐bp (g.30772543_30772566del24) deletion of CCDC157 were identified in five sporadic OAT patients, and 2 cases within one pedigree. In a mouse model, loss of *Ccdc157* led to male sterility with OAT‐like phenotypes. Electron microscopy revealed misstructured acrosome and abnormal head–tail coupling apparatus in the sperm of *Ccdc157*‐null mice. Comparative transcriptome analysis showed that the *Ccdc157* mutation alters the expressions of genes involved in cell migration/motility and Golgi components. Abnormal Golgi apparatus and decreased expressions of genes involved in acrosome formation and lipid metabolism were detected in *Ccdc157*‐deprived mouse germ cells. Interestingly, we attempted to treat infertile patients and *Ccdc157* mutant mice with a Chinese medicine, Huangjin Zanyu, which improved the fertility in one patient and most mice that carried the heterozygous mutation in *CCDC157.* Healthy offspring were produced. Our study reveals CCDC157 is essential for sperm maturation and may serve as a marker for diagnosis of OAT.

## 1 INTRODUCTION

Oligoasthenoteratozoospermia (OAT), obstructive azoospermia (OA), and nonobstructive azoospermia (NOA), together represent a significant proportion of clinical cases relating to male infertility. In the clinical management of male infertility, an array of procedures including detailed medical history and physical examination, semen analysis, hormonal assessment, imaging studies, testicular biopsies and Y chromosome microdeletion is currently the main criteria used for diagnosis.^1^, ^2^ Over recent years, genetic tests including whole‐exon sequencing (WES) and whole‐genome sequencing (WGS) have been applied for the screening of gene variants responsible for male infertility.^3^ Overall, about 15%–30% of male infertility cases have been considered to be due to genetic disorders.^4^, ^5^, ^6^ Within 2300 genes potentially involved in spermatogenesis,^7^ only about 38 genes have been identified to be related to NOA.^8^ The mechanisms of many of these genes involved in NOA or OAT remain largely unknown.

Spermatogenesis is a highly regulated and complex process which can be divided into three stages: spermatogonial mitosis, meiosis of spermatocytes and spermiogenesis.^4^ During spermatogenesis many aspects of cellular morphology and the overall order of developmental processes seem to occur similarly across different species.^9^, ^10^, ^11^ The acrosome is a Golgi‐derived cap‐like structure that stores hydrolytic enzymes.^12^ The acrosome–acroplaxome complex is an essential structure during spermatid development. It anchors to the anterior nuclear membrane by the acroplaxome which consists of a filamentous actin (F‐actin)‐and keratin‐containing plate with a desmosome‐like marginal ring.^13^ Caudally to the acrosome–acroplaxome is the manchette, a transient microtubule/F‐actin‐containing structure that shapes the developing spermatid head^14^ and acts as a conduit for transport proteins via molecular motor proteins to the head–tail coupling apparatus (HTCA).^15^ The HTCA acts as the anchor between the head and flagellum of the sperm and it is essential for sperm movement and fertilization. The axoneme forms the core of the flagellum, which is composed of a central pair of microtubules, structural elements such as radial spokes, and nine outer doublet‐microtubules (9 + 2).^16^ During acrosome biogenesis, numerous granules from trans‐Golgi stacks form and then accumulate in the concave region of the nuclear surface, fusing into a large acrosome granule.^17^ Many Golgi‐associated proteins have been identified to be involved in this process, including GOLGA4,^18^ GOPC,^19^ GOLGA3,^20^ and GM130.^21^

The coiled‐coil domain‐containing (CCDC) family of proteins have a conserved coiled‐coil motif that is responsible for molecular recognition and protein refolding.^22^ Many members of the CCDC family are highly and specifically expressed in testis, playing explicit roles in spermatogenesis. Among these, *CCDC65* is known to be expressed in the flagella of human sperm and is required for sperm capacitation^23^; *Ccdc172* localizes in the middle piece of spermatozoa, predominantly concentrated at the mitochondrial sheath of the flagella^24^; and *Ccdc181* ^25^ localizes in the spermatic flagella and interacts with HOOK1, along with *CCDC189*,^26^ *Ccdc42*,^27^ *CCDC34* ^28^ and *CCDC9*,^29^ which function together to maintain the structure and function of sperm flagella. *CCDC65*,^30^ *CCDC39*,^31^ *CCDC40* ^31^, ^32^ and *CCDC151* ^33^ are important for motile cilia function. Other members such as *Ccdc62*,^34^ *Ccdc136* ^35^ and *Ccdc87* ^36^ have been reported to influence the sperm head where *Ccdc62* specifically localizes at the acrosome in mature sperm^34^; and *Ccdc136* and *Ccdc87* are responsible for the acrosome formation and fertilization in mice. *CCDC157* is also highly expressed in the testes from *Drosophila* to mammals.^37^ Bioinformatics database analysis suggests that *CCDC157* expression is significantly decreased in NOA patients.^37^ An in vitro study has reported that CCDC157 is localized on Golgi membranes along the secretory pathway and interacts with resident proteins of ER‐Golgi membranes.^38^

In this study, we identified five sporadic cases and one pedigree of OAT as associated with the *CCDC157* mutation. To investigate the function of *CCDC157* involved in spermatogenesis, we then established a mouse mutant and confirmed that *CCDC157* defects in mice also resulted in male sterility. In such mice the most obvious phenotypic defects involved the sperm acrosome and sperm motility. Further studies indicated CCDC157 dysfunction lead to abnormal Golgi morphology and down regulation of both acrosome formation‐associated genes and lipid metabolism genes. In addition, *Ccdc157* ^*−/+*^ male mice show delayed reproductive age. Interestingly, upon application of a Chinese medicine, Huangjin Zanyu, as medical treatment for patients or for mouse heterozygous mutants, reproductive ability was improved. Our findings demonstrate that CCDC157 is required for acrosome formation and has potential as a molecular target for the diagnosis of OAT.

## 2 RESULTS

### 2.1 Identification of *CCDC157* variants in the sporadic cases and a pedigree of OAT

**FIGURE 1:**
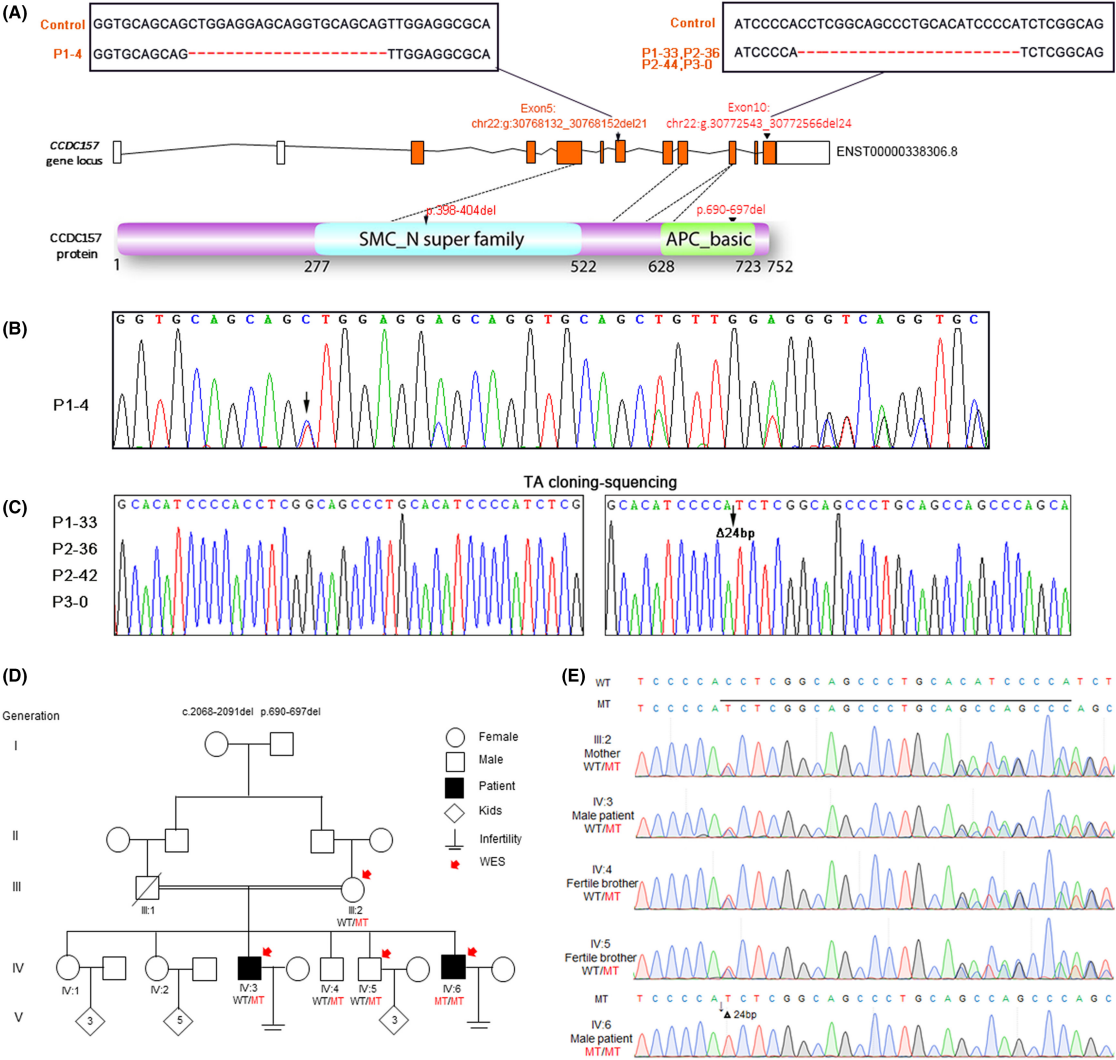
*CCDC157* deficiency in sporadic cases and in a pedigree of OAT. (A) The schematic diagrams of human CCDC157 gene loucs and protein domain structure. Blue colour shows the SMC_N super family domain and green colour shows the APC_basic domain. The patient (P1‐4) contains a deletion of 21 bp at the position 15,520–15,540 (Chr22:g:30768132_30768152del21, GRCh37). The other patients (P1‐33, P2‐36, P2‐42 and P3‐0) carry one copy of a deletion of 24 bp (g.30772543_30772566del24, GRCh37). (B) Chromatograms of the *CCDC157* from the patient (P1‐4) and the black arrow indicates the double peak. (C) Analysing of CCDC157 deficiency in four cases of with CCDC157 deficiency. The PCR products templated from the patients (P1‐33, P2‐36, P2‐42 and P3‐0) DNA were ligated with a T‐vector. Single clones were then sequenced and shown to carry one copy of the CCDC157 mutation (g.30772543_30772566del24). Black triangles indicate the 24 bp deletion at exon 10 and the 8 amino acids deletion (p.690‐697) at the APC_basic domain. (D) The pedigree with male infertility from a consanguineous family. Four individuals, indicated by red arrows, were selected for whole‐exome sequencing. The double horizontal lines indicate consanguineous marriage. Slashes represent deceased family members. Open symbols = unaffected; filled symbols = affected; diamond = children, unknown sex; WT wildtype, MT mutant. (E) Chromatograms of the CCDC157 non‐frameshift deletion (g.30772543_30772566del24) in genomic DNA from all the available family members.

With permission, we performed screening of 92 patients associated with OAT phenotypes in the Women's Hospital of Zhejiang University School of Medicine. Any patients with other health issues such organic lesions of the reproductive system, untreated endocrine disorders, drug or alcohol abuse within recent 2 years, chromosomal abnormalities, AZF abnormalities, cryptorchidism or mumps were excluded from the study. Their spouses all had normal ovulatory cycle and normal hysterosalpingography. We carried out PCR and sequencing experiments in these samples. Primers were designed to mainly target the two conserved domains of the CCDC157, SMC_N super family domain and the APC_basic domain.^37^ Five out of the 92 OAT patients were identified to carry one copy of a *CCDC157* gene defect. Sequencing results showed that P1‐4, a 34‐year‐old man, had a *CCDC157* mutation containing a non‐frameshifting deletion of 21 bp at the position 15,520–15,540 of this gene (g.30768132_30768152del21), leading to the deletion of 7 amino acids (Figure 1A). Sequencing of four other patients (P1‐33, P2‐36, P2‐42 and P3‐0) revealed CCDC157 mutations containing a non‐frameshifting deletion of 24 bp (g.30772543_30772566del24) in genomic DNA within the coding region of the *CCDC157* gene, which had led to the deletion of 8 amino acids (Figure 1B,C).

In searching for genetic causes of male infertility, we also focused on a consanguineous Pakistani pedigree linked to a *CCDC157* mutation. Two infertile brothers, IV:3 (married for 22 years) and IV:6 (married for 20 years) were diagnosed with mild and severe OAT, respectively. These two affected brothers, born to a first‐cousin marriage (Figure 1D), had a normal 46 and XY karyotype, without any reported history of tumour/cancer, drinking or smoking. The third brother IV:5 had a child after 8 years of marriage (Figure 1D). The fourth brother IV:4 was diagnosed with night blindness and did not get married. The clinical findings of the most severe OAT brother IV:6 are summarized in Table S1. In addition, we also identified another 12 individuals out of 739 NOA or OAT patients carrying the same heterozygous non‐frameshifting deletion (g.30772543_30772566del24) in the genomic DNA of the CCDC157 gene in our clinical collection of cases. In the Genome Aggregation Database (gnomAD) database, the frequency of the 21 bp deletion variant was 0 among the East Asian and 0.002557% among the total population. The frequency of the 24 bp deletion variant was 0.01006% among the East Asian, 0.001772% among the total population (http://gnomad‐sg.org/).

WES of patients IV:3, IV:6, fertile brother IV:4 and IV:5 as well as their mother (III:2) showed more than 400,000 variants per individual, covering at least 99% of the targeted sequence at 90× or greater depth. Considering that the individuals were born to a consanguineous marriage, variants following autosomal recessive inheritance were prioritized. These variants were filtered using a series of criteria. We then noted a 24 bp deletion in *CCDC157* gene. To evaluate the transmission pattern of the variant in the family, we performed targeted Sanger sequencing in the available family members (III:2, IV:3, IV:4, IV:5 and IV:6). Results showed that III:2, IV:3, IV:4 and IV:5 all were heterozygous with a double peak and that IV:6 was homozygous with single peak (Figure 1E). As the CCDC157 protein contains two conserved domains, the SMC_N super family domain and the APC_basic domain, we analysed the mutation position and found that it occurred in exon 10 and caused a 24 bp deletion (g.30772543_30772566del24) at cDNA from 2068 to 2091 leading to a non‐frameshifting deletion of 8 amino acids from 690 to 697 in the APC_basic domain (Figure 1C). This was completely identical to the mutation we found in the above highlighted Chinese cases. We calculated that the frequency of the 21 bp deletion variant in our collected OAT clinic cases was 0.5434783%; and the frequency of the 24 bp deletion variant was 1.3237064%, which was far higher than the corresponding values in the general population. Thus, we considered that, for these cases, *CCDC157* was also a candidate gene responsible for OAT, causing male infertility in this family.

### 2.2 Modelling of *Ccdc157* loss‐of‐function mutant allele in mice

**FIGURE 2:**
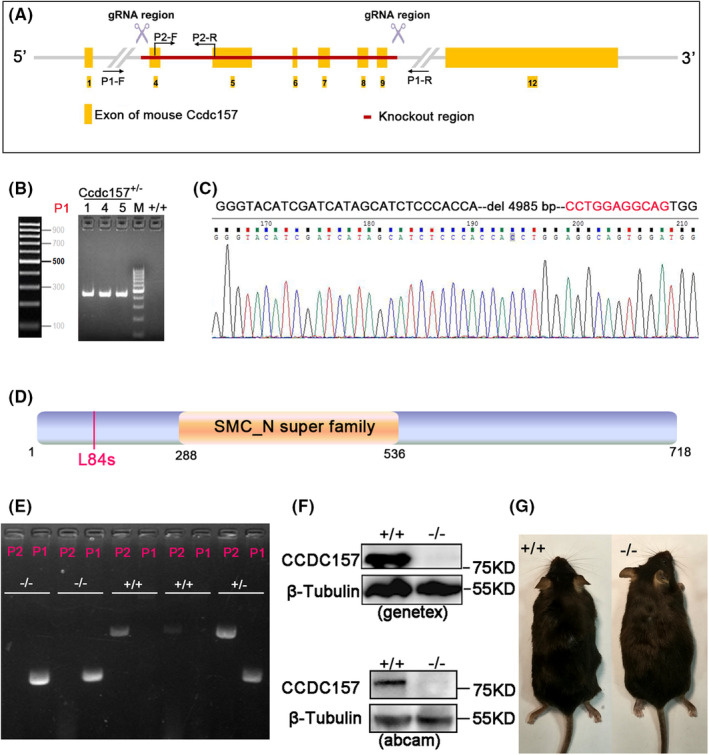
Generation and identification of *Ccdc157* mutants. (A) Schematic representation of the *Ccdc157* locus. Yellow tetragonal shapes indicate exons. The red line indicates the region between two gRNAs. P1 and P2 are two primers pairs designed to identify the results of the CRISPR/Cas9 analysis. (B) PCR analysis of the first filial generation mice. Two female animals (Lane 4 and 5) and one male mouse (Lane 1) were identified with knockout of the red line region of *Ccdc157*. (C) Graph showing the sequencing information of the genomic data of the first filial generation indicating that a deletion of 4985 bp and an insertion of additional 11 bp had occurred in these animals. (D) Schematic diagrams of mouse CCDC157 protein domain structure. L84s indicates that the mutation allele has resulted in an 84th leucine followed by a stop codon. (E) Genomic DNA PCR genotyping of wild‐type (WT) (*Ccdc157* ^*+/+*^), heterozygous (*Ccdc157* ^*+/−*^), and knockout (*Ccdc157* ^*−/−*^) mice. (F) Western Blot analysis of the testes extracts from *Ccdc157* ^*+/+*^and *Ccdc157* ^*−/−*^. Lysates were probed with two anti‐CCDC157 antibodies and anti‐tubulin antibody. (G) WT control (*Ccdc157* ^*+/+*^) mouse next to a knockout (*Ccdc157* ^*−/−*^) sibling shows no obvious phenotype in the mutants.

To investigate the mechanism of the mutation of *CCDC157* involved in male infertility, we employed CRISPR/Cas9 to generate *Ccdc157* mutants in mice. The two gRNAs were designed to flank the exon 4 and the exon 9 region (Figure 2A). After several steps including cell transfection, FACS, injection, transplantation, we obtained the founder mice. For genotyping of *Ccdc157* in the founder mice, genomic DNA from the tails of newborn mice was extracted and PCR experiments carried out. Three mice containing a deletion allele of *Ccdc157* were identified (Figure 2B). We then sequenced the deletion allele of *Ccdc157* and found that there was a deletion of 4985 bp and an insertion of an additional 11 bp allele (Figure 2C), leading to a truncated CCDC157 protein with the 84th amino acid (leucine) followed by a stop codon (Figure 2D). Heterozygous *Ccdc157* mutant mice were bred and screened for homozygous *Ccdc157* mutant offspring. The genotypes of the offspring were identified by PCR (Figure 2E).

Using two CCDC157 poly‐antibodies, one recognizing the 141–261 amino acids of CCDC157 (Abcam) and another recognizing 431–480 amino acids of CCDC157 (Gentex), we tested whether the generated *Ccdc157* mutants were null alleles. Western blot showed that in wild‐type (WT) testis extracts, both antibodies against CCDC157 were recognized by a band of about 80 kDa, while in the *Ccdc157*‐null mice testis extracts this band was absent (Figure 2F). In addition, qPCR and western blot showed that the expression levels of *Ccdc157* had dramatically decreased in *Ccdc157*‐null mice, with downregulated *Ccdc157* expression also detected in *Ccdc157* ^*+/−*^ mice (Figure S1A). We tracked the development of the *Ccdc157* ^*−/−*^ mice and observed no differences in body phenotype between *Ccdc157* ^*−/−*^ mice and their WT littermates (Figure 2G). These data confirmed the *Ccdc157* mutant to be a null allele and that *Ccdc157* deficient mice are viable with no defects in growth and development.

### 2.3 *Ccdc157*‐deficient male mice were characterized by OAT‐like phenotypes

To test the fertility outcomes, *Ccdc157* ^*−/−*^ male mice were crossed with WT females and showed copulation behaviour, with the vaginal plugs observed in females. However, no females became pregnant (Figure S1B), suggesting that *Ccdc157* ^−/−^ males were sterile. In addition, *Ccdc157* ^*+/−*^ males exhibited a delayed reproductive age by about 28–30 days. WT males average took 29 days before WT females gave their first births after cohabiting, whereas *Ccdc157* ^*+/−*^ male mice took an average of 58 days before *Ccdc157* ^*+/−*^ or *Ccdc157* ^*−/−*^ females gave their first births (Table S2). *Ccdc157* ^*+/−*^ or *Ccdc157* ^*−*/−^ females cohabiting with *Ccdc157* ^*+/−*^ males also exhibited repeated offspring consumption and other aggressive parental behaviour, resulting in difficulties in obtaining live litters.

**FIGURE 3:**
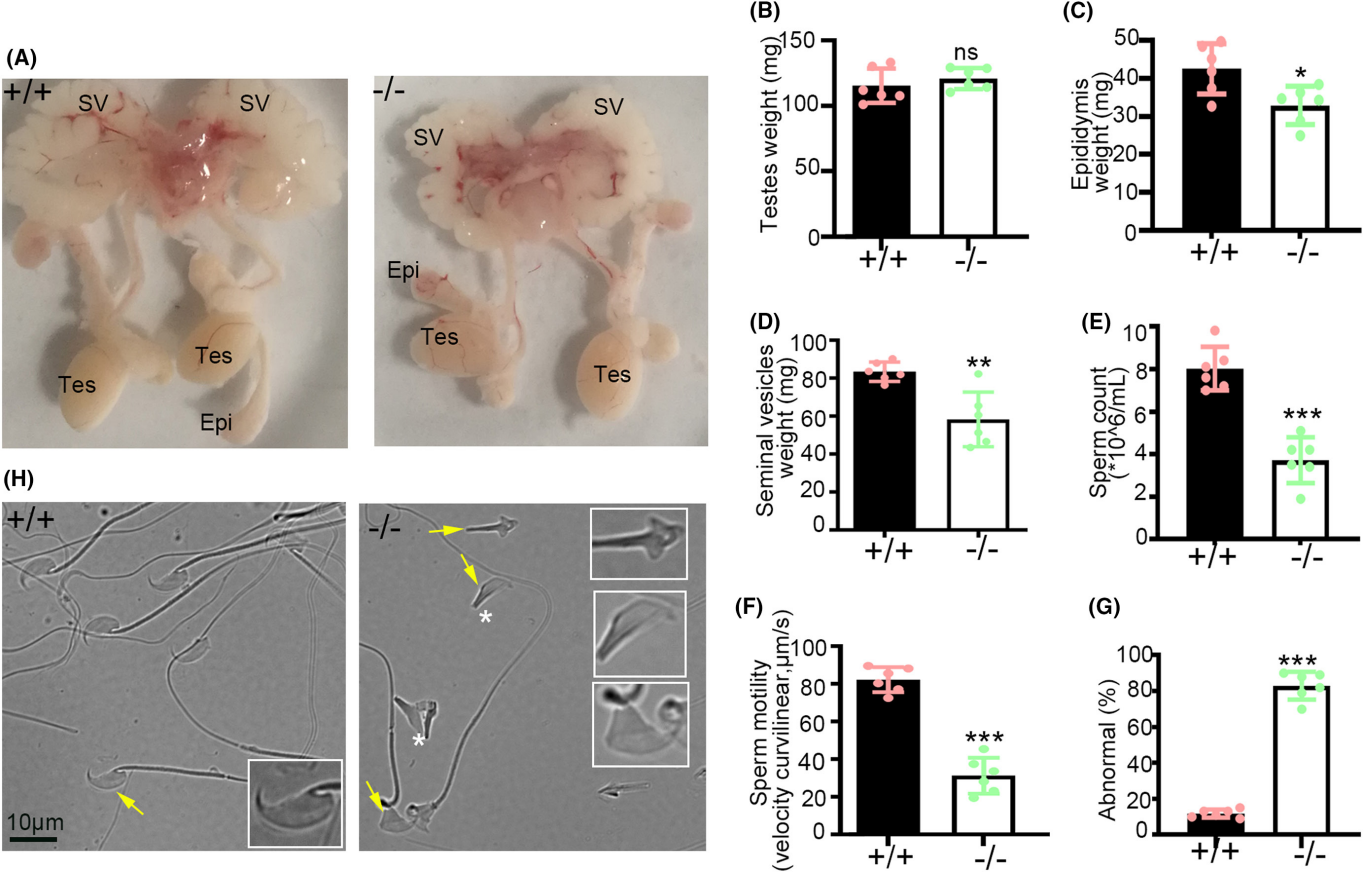
Knockout *Ccdc157* leads to oligoasthenoteratospermia‐like phenotype. (A) Images of dissected male genitalia from 8‐week‐old C57BL/6, and *Ccdc157* ^−/−^; Tes, testis; Epi, epididymis; SV, seminal vesicle. (B) Testis weight. (C) Epididymis weight. (D) Seminal vesicle weight. (E) Concentrations of epididymal sperm. (F) Sperm motility. (G) Percentage of epididymal sperm showing abnormal head. (H) Microscopic images of epididymal sperm. Six mice of each genotype were used in this phenotypic statistical analysis in this study. Data are presented as mean ± SD. *p < 0.05, **p < 0.01, ***p < 0.001.

We examined the reproductive system, including the testis, epididymis, and seminal vesicles, all of which appeared normal in *Ccdc157* ^*−/−*^ male mice (Figure 3A). Quantitative analysis demonstrated no significant difference in testes size and weight between *Ccdc157* ^*−/−*^ male mice and WT mice (Figure 3B). However, the weight of the epididymis and seminal vesicles from *Ccdc157* ^*−/−*^ mice was decreased compared to those from WT mice (Figure 3C,D), probably due to less sperm produced in *Ccdc157* ^*−/−*^ mice than those in WT mice.

Further analysis revealed a significant decrease in sperm count and motility in caudal epididymis of *Ccdc157* ^*−/−*^ mice compared to WT mice (Figure 3E,F). Microscopic analysis of sperm morphology showed a high incidence of malformed sperm heads (87.3%) in *Ccdc157* ^*−/−*^ mice compared to WT mice (10%), with frequent sperm decapitation also observed in *Ccdc157* ^*−/−*^ mice (Figure 3G,H). There were no significant differences in the shape and length of sperm flagella between WT and *Ccdc157* ^*−/−*^ mice (Figures 3H and S2A,B). In addition, we observed compromised sperm motility and higher rates of malformed sperm heads in *Ccdc157* ^*+/−*^ mice than WT mice (Figure S2C). Taken together, our findings suggest that the phenotype of *Ccdc157* ^*−/−*^ male mice strongly resembles that of OAT patients observed in a clinical setting, with significant impairments in sperm count, motility and morphology.

### 2.4 The *Ccdc157*‐null mutation affects acrosome formation and sperm nuclear shaping

**FIGURE 4:**
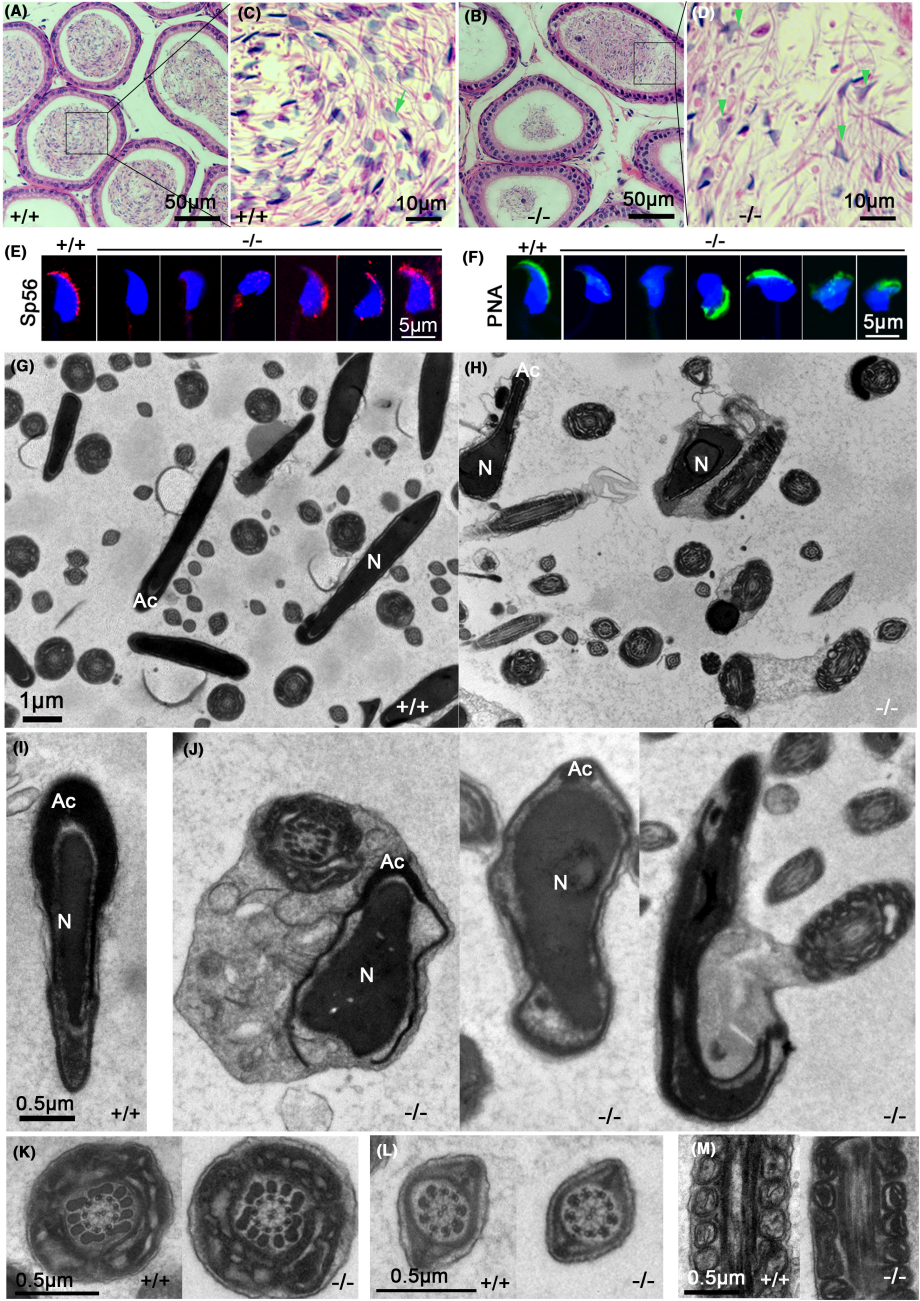
Structural analyses of spermatozoa in *Ccdc157*‐null mutants. (A–D) Haematoxylin and eosin stained images. (A) wild‐type (WT) epididymis, (B) *Ccdc157*‐null mutant epididymis; (C, D) Higher magnification images of figure A and B; green arrow indicates sickle‐shaped sperm head. Green triangles indicate non‐sickle‐shaped sperm heads. Sp56 (E) and PNA (F) staining of mature spermatozoa in epididymis from WT animals and *Ccdc157*‐null mutants. Nuclei were stained with DAPI. (G, H) TEM figures of epididymis from WT animals and *Ccdc157*‐null mutants. (I, J) TEM images of epididymal sperm heads. The nuclei of WT mature sperm (I) is elongated and covered with a cap‐like acrosome, while the heads of the *Ccdc157*‐null mutants (J) include distorted or detached acrosomes, irregular nuclei, and cytoplasmic/membranous organelle residue. N, nucleus; Ac, acrosome. (K) TEM images of cross sections of Step 15 spermatid flagella at the midpiece show the outer dense fibrous structural similarities between WT animals and *Ccdc157*‐null mutants. (L) TEM images of cross section of Step 15 spermatid flagella at the principal piece. (M) TEM analysis of mitochondria along the midpiece of Step 15 spermatids from WT and *Ccdc157*‐null mutants.

Sections of the cauda epididymis with haematoxylin and eosin (HE) staining showed that there were markedly fewer sperm in the lumens of the epididymal ducts of *Ccdc157*‐null mice than that of the WT siblings (Figure 4A,B). A higher magnification view showed that most of sperm of mutants appeared with non‐sickle shaped heads (Figure 4C), in contrast to those of WT mice (Figure 4D). To examine whether the absence of CCDC157 resulted in impairment to the acrosome, we used two acrosome‐specific markers, Sp56 (ZP3R) and peanut agglutinin (PNA), for immunofluorescence staining. In WT mice, most spermatozoan acrosomes showed a typical crescent shape on the head of the sperm (Figure 4E,F, left panel); while in *Ccdc157*‐deficient mice, spermatozoan acrosomes in the cauda epididymis exhibited various defective shapes, including deformation, mis‐localization and fragmentation (Figure 4E,F, right panel). TEM observations also showed the irregular shaped acrosome and nuclei in the existing small number of sperm in the epididymal duct of *Ccdc157* ^*−/−*^ mice (Figure 4I), as compared to a high regularity in shape for the WT mice (Figure 4G). The nuclei of the mature sperm were elongated and covered with a cap‐like acrosome in the WT (Figure 4I). By contrast, distorted or detached acrosomes and irregular nuclei, some with remaining cytoplasm/membranous organelle residue, were found in the heads of the *Ccdc157*‐null mice (Figure 4J). *Ccdc157* loss seemed not to affect sperm flagella which presented with the typical arrangement of nine outer microtubule doublets surrounding the central pair (9 × 2 + 2) (Figure 4K–M). Similarly, no ultrastructural abnormalities of axoneme (Figure 4K–M), mitochondria (Figure 4M), outer dense fibres (Figure 4K) or fibrous sheath (Figure 4L) were apparent for *Ccdc157* ^*−/−*^ sperm. These observations suggest that *Ccdc157* plays a particular and critical role in nuclear shaping and acrosome formation.

### 2.5 HTCA and acroplaxome–acrosome formation were abnormal in Ccdc157‐deficient male mice

**FIGURE 5:**
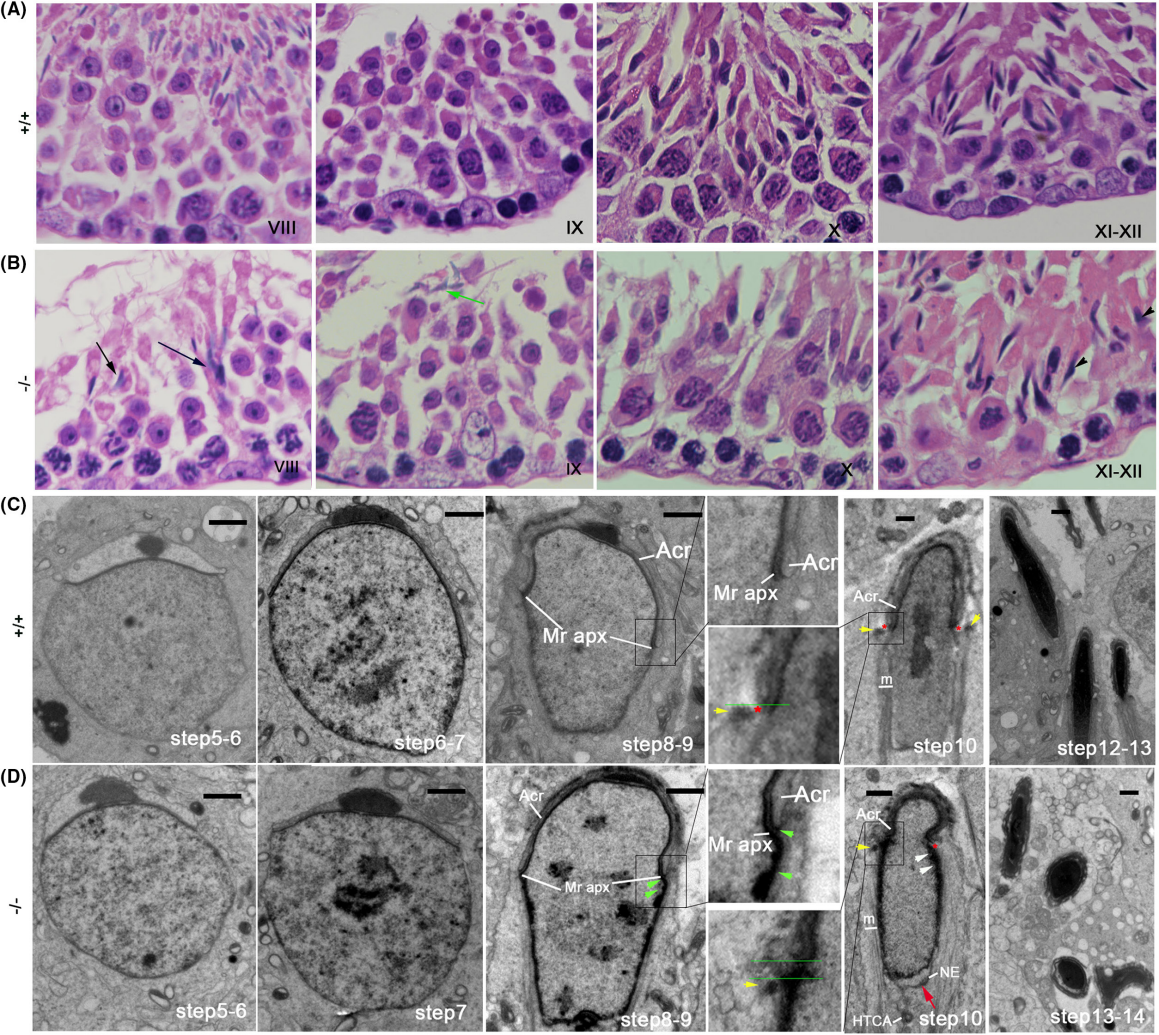
*Ccdc157* efficiency affects HTCA and acroplaxome–acrosome formation in mice. HE‐stained sections of seminiferous tubules from *Ccdc157* ^*+/+*^ (A) or *Ccdc157*‐null mutant (B) at Stages VIII–XII of spermatogenesis. Black arrowheads indicate the heads of representative elongating spermatids (Steps 9–12, respectively). Black arrows and arrowheads show abnormal head sharp of spermatozoa in *Ccdc157* ^*−/−*^ mice. Green arrows indicate spermatozoa attached to Sertoli cells. (C, D) TEM images of Steps 5–13 spermatids from *Ccdc157* ^*+/+*^ or *Ccdc157* ^*−/−*^ mice. m:manchette; Acr: acrosome; Mr apx: marginal ring of the acroplaxome; HTCA, head–tail coupling apparatus; NE: nuclear envelope. Green arrowheads label the unclear acroplaxome marginal ring. The red star shows the groove belt region of Step 10 spermatids.Red arrow indicates the nuclear envelope as detached from condensed chromatin. Yellow arrowheads point to the perinuclear rings of the manchette. Green line in figure C shows the posterior edge of the acrosome is parallel with the perinuclear rings. Green lines in figure D shows the posterior edge of the acrosome is mispositioned, not being juxtaposed to the perinuclear rings. Scale bars: 1 μm.

Testis sections and HE staining of WT samples in mice showed that, according to Stages I–XII of the seminiferous epithelial cycle,^39^ mature spermatozoa should be released into the seminiferous lumen by stage VIII (Figure 5A). However, in *Ccdc157* ^*−/−*^ mutant mice, even at the Stages VIII–IX of the seminiferous epithelial cycle some of the spermatozoa remained attaching to Sertoli cells (Figure 5B, arrows). Many other morphological abnormalities (Figure 5B, arrowheads) were also noted. This indicated that some of the spermatozoa in *Ccdc157* ^*−/−*^ mice had undergone delayed spermiation, leading to the lower sperm counts observed above.

During acrosome biogenesis, four different phases (together incorporating 16 steps), have been identified in the mouse. These are the Golgi phase (Steps 1–3), Cap phase (Steps 4–7), acrosome phase (Steps 8–12) and maturation phase (Steps 13–16).^17^ TEM observations revealed that the Cap phase (Steps 4–7) of *Ccdc157* ^*−/−*^ mice appeared normal with round shaped spermatids (Figure 5C). However, at Step 8, as spermatids begin to elongate (the acrosome phase), ambiguous marginal rings of the acroplaxome (Mr apx) appeared in the spermatids of *Ccdc157* ^*−/−*^ testes (Figure 5D, green arrows), associated with the aberrant head structures which were then universally presented by Step 10. In these *Ccdc157* ^*−/−*^ spermatids, the nuclear envelope (NE) were detached from condensed chromatin (Figure 5D, red arrow), and the head‐tail coupling apparatus (HTCA) was separated from the nucleus (Figure 5D, Step 10). This may explain the sperm decapitation and disrupted sperm motility occurring in *Ccdc157* ^*−/−*^ mice. In addition, an obviously malformed groove belt region (Figure 5D, red star) and deformed underlying nuclear lamina (Figure 5D, white arrows) were observed in *Ccdc157* ^*−/−*^ mice. Similarly, the posterior edge of the acrosome was mispositioned, not being juxtaposed to the perinuclear rings of the manchette (Figure 5D, green lines) in *Ccdc157* ^*−/−*^ mice, while occurring as parallel with the perinuclear rings in the WT mice (Figure 5C, green line). These results indicate that *Ccdc157* is important for the acroplaxome–acrosome formation of spermatids.

### 2.6 *Ccdc157* deficiency alters the transcriptome of mouse testis

**FIGURE 6:**
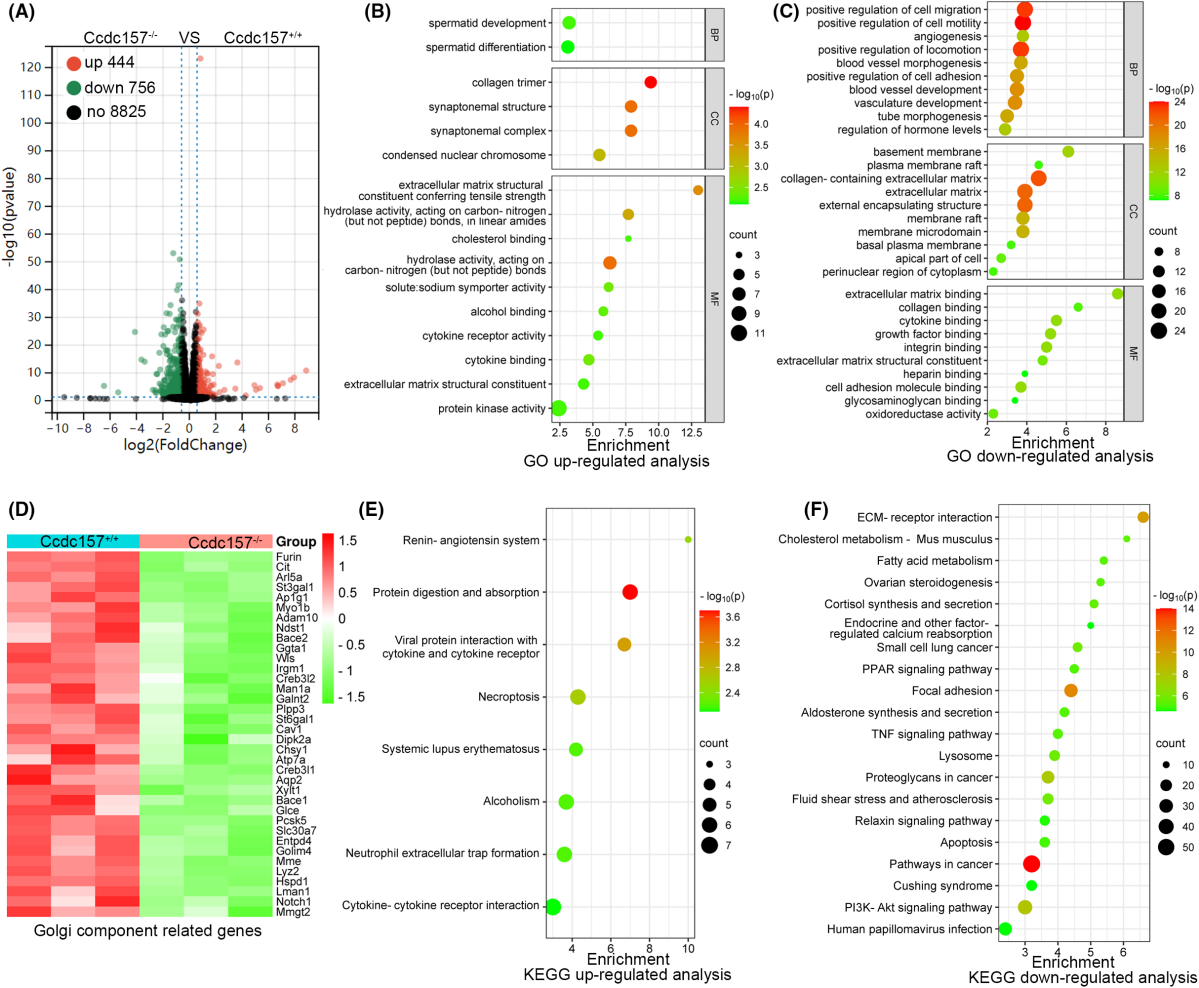
Transcriptome analysis of the testes of *Ccdc157* ^*−/−*^ and WT mice. (A) Volcano plot showing 1200 DEGs from *Ccdc157* ^*−/−*^ and WT group (*n* = 3 per group). Red dots represent upregulated genes and green dots downregulated genes. GO functional analysis for upregulated DEGs (B) and downregulated DEGs (C) in *Ccdc157* ^*−/−*^ versus WT mice were grouped into different functional categories: biological process (BF), cellular component (CC) and molecular function (MF). (D) The heat map of Golgi component‐related genes in *Ccdc157* ^*−/−*^ and WT mice. Representative dot plot of top 10 significantly enriched KEGG pathways for downregulated (E) and upregulated genes (F).

In order obtain novel insights into the molecular mechanisms underlying *Ccdc157* regulation of spermatogenesis, we performed RNA‐Seq to uncover the differences in the testis transcriptomes between *Ccdc157* mutant and WT mice. PCA analysis revealed clustering of three biological replicates in each group which clearly demonstrated the differences between *Ccdc157* mutant and WT (Figure S3). Comparative analysis showed a total of 1200 differentially expressed genes (DEGs) for the *Ccdc157* mutant as compared with the controls, with a cutoff at 1.5‐fold and a false discovery rate of <0.05 (Table S3). We identified 756 genes that were significantly downregulated and 444 genes that were significantly upregulated in the testes of the *Ccdc157* mutants, as shown in the Volcano plot in Figure 6A. To further analyse and classify the biological function of DEGs, we performed Gene Ontology (GO) enrichment analysis, which was sub‐divided into three categories: biological process (BP), cellular component (CC) and molecular function (MF) (Tables S4 and S5). Upregulated gene‐enriched GO terms were identified and are shown in Figure 6B and the Top 10 downregulated gene‐enriched GO terms are presented in Figure 6C. In the BP group, the upregulated DEGs were those mainly enriched in spermatid development and spermatid differentiation stages (Figure 6B), while downregulated DEGs were those mainly enriched in the positive regulation of cell migration/motility and in aspects of cell adhesion and regulation of hormone levels (Figure 6C). In the CC group, Golgi cisterna, Golgi stack and Golgi apparatus subcompartment‐related genes showed as significantly downregulated in *Ccdc157* mutant testes (Figure 6D, Table S6).

Kyoto Encyclopedia of Genes and Genomes (KEGG) enrichment analysis revealed that the genes upregulated in *Ccdc157*‐deficient testes were those associated with renin‐angiotensin system, necropotosis and the cytokine‐cytokine receptor interaction (Table S7). KEGG analysis also showed genes significantly downregulated in *Ccdc157*‐deficient mice involved in ECM‐receptor interaction, cholesterol metabolism, fatty acid metabolism, apoptosis and hormone biosynthesis (Table S8). Upregulated KEGG pathways and the top 20 KEGG downregulated pathways were identified by analysing significantly DEGs which are shown as dot plots (Figure 6E,F).

### 2.7 Mis‐localization of Golgi apparatuses and downregulation of acrosome formation‐ and lipid metabolism‐associated genes and in *Ccdc157* ^*−/−*^ testes

**FIGURE 7:**
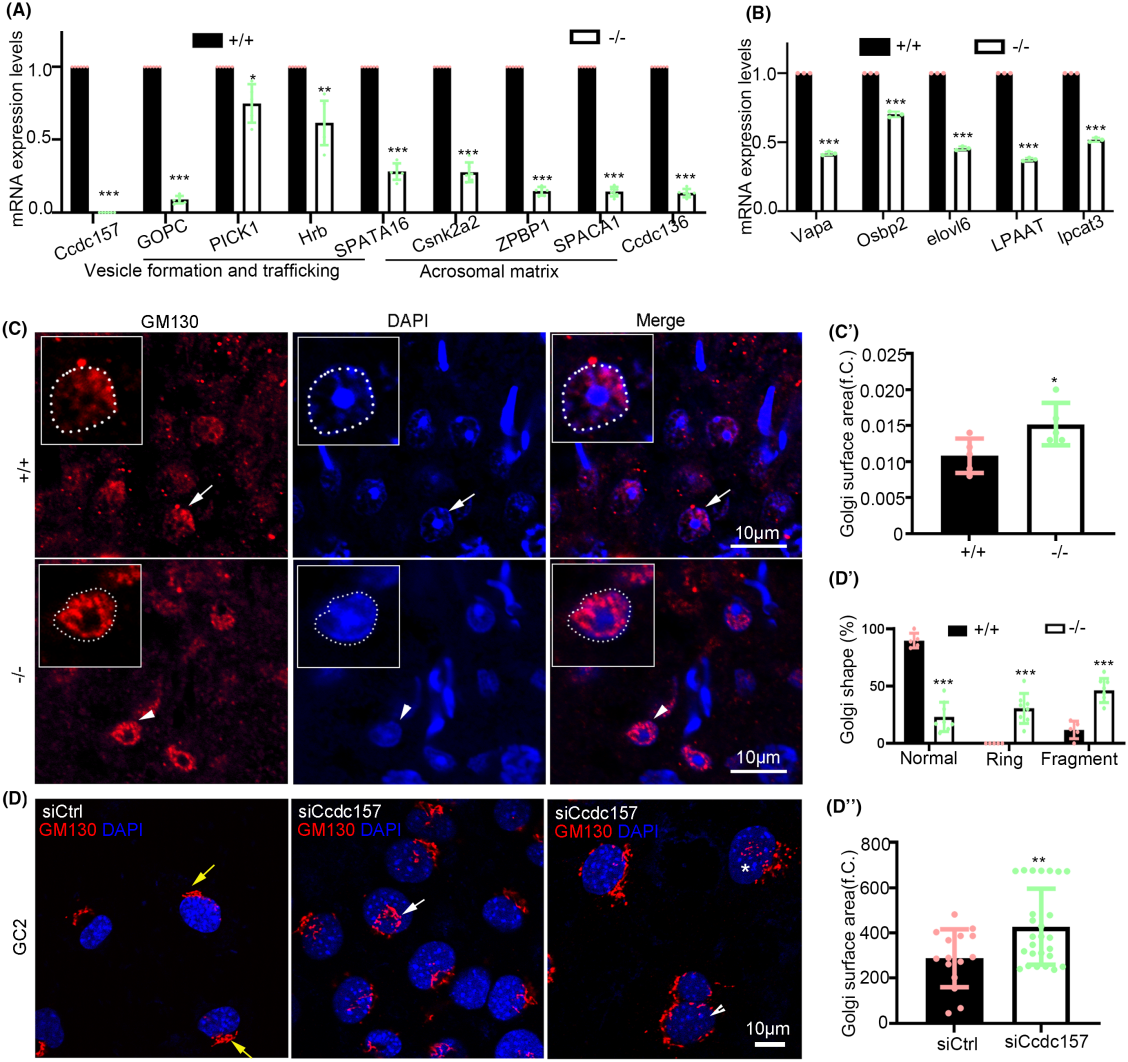
Mis‐localization of Golgi apparatuses and downregulation of acrosome formation‐ and lipid metabolism‐associated genes and in *Ccdc157* ^*−/−*^ testes. (A) RT‐PCR amplification of acrosome formation‐associated genes from testes of wild‐type (WT) and *Ccdc157* mutants with β‐actin used as a loading control. (B) RT‐PCR amplification of lipid metabolic genes from testes of WT and *Ccdc157* mutants with β‐actin used as a loading control. (C) *cis*‐Golgi membrane marker GM130 staining of sections of seminiferous tubules from WT or *Ccdc157*‐null mutants. The white arrow and arrowhead indicate the cells that are magnified and shown in the upper left boxes. (C′) Quantification of the percentage of Golgi surface area from WT or *Ccdc157*‐null testes. (D) GM130 staining of GC2 cells upon knock down of *Ccdcc157*. The yellow arrows indicate normal shaped Golgi, the white arrow indicates ring‐shaped Golgi, and the arrowhead and star indicate the fragmented Golgi. (D′) Quantification of the percentage of cells with different shaped Golgi complex in *Ccdcc157*‐depleted cells. (D″) Quantification of the percentage of Golgi surface area from *Ccdcc157*‐depleted cells and control cells.

As the acrosome formation proceeds, the sequential fusions of Golgi‐derived pro‐acrosomal vesicles form a mesh of cytoskeletal fibres as a cap overlying the nucleus.^17^ We examined the expression levels of related genes and proteins including the following: Ccdc136, involved in acrosome biogenesis^35^; GOPC,^19^ a protein interacting with C kinase 1 (PICK1)^40^; and Hrb,^41^ involved in vesicle formation and trafficking. We also looked at the acrosomal matrix genes SPATA16,^42^ Csnk2a2,^43^ ZPBP1^44^ and SPACA1.^45^ Results showed that the mRNA levels of all these genes, together with that of GOPC as well as Hrb protein levels were significantly down regulated in *Ccdc157* ^*−/−*^ testes (Figures 7A and S4), compared to the WT.

CCDC157 has been reported to be indispensable for the fusion of transport carriers with the Golgi complex in Hela cells.^38^ We examined Golgi apparatus in sperms of the WT and *Ccdc157* ^*−/−*^ mice by immunostaining of Golgi‐specific protein GM130, a *cis*‐Golgi maker.^46^ GM130‐positive punctae were detected as mainly accumulating at one side of the nucleus in the WT round spermatids. Interestingly, in the round *Ccdc157* ^*−/−*^ spermatids, the GM130‐positive punctae were distributed throughout the cytoplasm and surrounded the nucleus in a ring (Figure 7C). Quantification of the Golgi complex surface area indicated an increase in Golgi apparatus of *Ccdc157* ^*−/−*^ spermatids (Figure 7C′), compared to that of WT. To further confirm, we performed knockdown of Ccdc157 with siRNA using a mouse pachytene spermatocyte‐derived cell line, GC‐2.^47^ In the GC‐2 cells lacking *Ccdc157*, the morphology of Golgi was categorized into three groups, including normal localization, ring shaped localization and fragmented distribution. Abnormal ring‐shaped Golgi architecture (Figure 7D′, white arrow) and fragmented Golgi (Figure 7D′, arrowhead and star) were observed in GC‐2 cells upon *Ccdc157* knockdown. The surface area of GM130‐indicated Golgi membrane was also increased compared to that of control cells (Figure 7D″). We performed co‐immunostaining of CCDC157 and GM130 and detected partial co‐localization of the signals of CCDC157 and GM130 on the Golgi apparatus in mouse sperm (Figure S5).

As we had previously found that the *Drosophila* homologue of CCDC157, *Pif1A*, played a potential role in regulating lipid metabolism‐related genes during spermatogenesis,^37^ we wondered if this is also the case for mice. Analysis of the homologues of those lipid metabolism‐related genes, including *Vapa, Osbp2, Elovl6, LPAAT* and *Lpcat3* in mice testes, showed that the mRNA levels of all these genes were also down regulated in *Ccdc157* ^*−/−*^ testes (Figure 7B). Particularly notable was Osbp2, as a sterol‐binding protein of the oxysterol‐binding protein (OSBP)‐related protein family, which has previously been reported to activate sphingomyelin synthesis in the Golgi complex.^48^ Deficiency of Osbp2 has been shown to cause male infertility with a corresponding severe OAT phenotype in mice.^49^ Our observations suggested that CCDC157 might act as an important regulator interplaying with the acrosome biogenesis and lipid metabolism‐related genes and therefore control the Golgi function and acrosome formation during spermiogenesis.

### 2.8 The Huangjing Zanyu Chinese medicine could restore reproductive ability caused by CCDC157 defects in clinical patients and in mice

**FIGURE 8:**
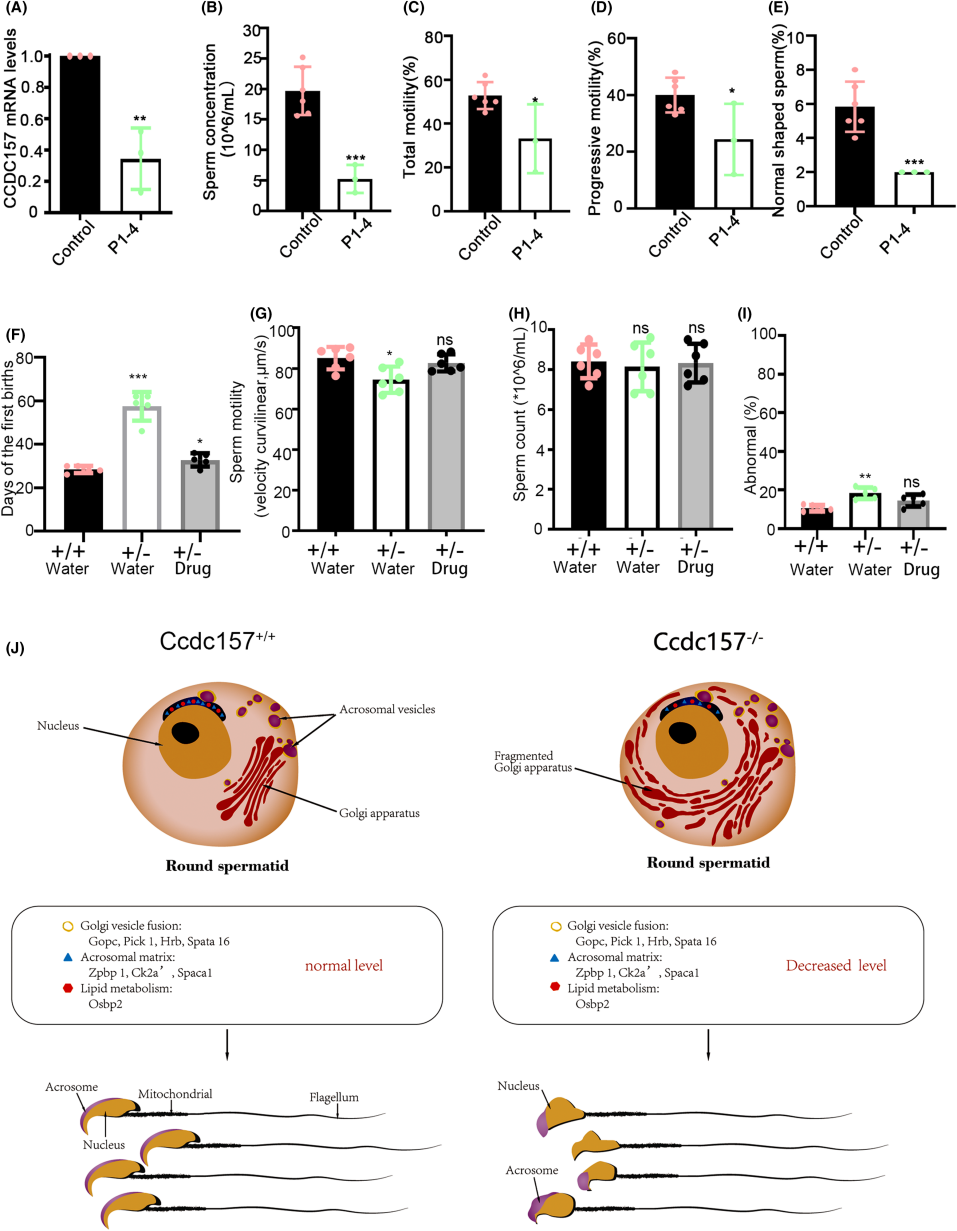
The Huangjing Zanyu Chinese medicine could restore reproductive ability caused by some CCDC157 defects in clinical patients and in mice. (A) Transcription expression levels of *CCDC157* in the patient (P1‐4)'s blood compared with that of *CCDC157* intact men. (B) Sperm concentrations. (C) Total motility. (D) Progressive motility. (E) Percentage of normal shaped sperm. (F) Average days for the females gave their first births after cohabiting with the male mice treated with Huangjing Zanyu or water. The *Ccdc157* ^*+/−*^ mice in the drug group took an average of 33 days to give births for the first time after cohabiting with males, while the *Ccdc157* ^*+/−*^ mice in the control group took an average of 58 days for their first births. (G) Sperm motility. (H) Sperm concentrations. (I) Percentage of abnormal shaped sperm. (J) A graphic illustration of CCDC157 function in the regulation of Golgi apparatus and acrosome formation during spermiogenesis.

Many traditional Chinese medicines have been shown provide benefits in the enhancement of sperm quality including sperm count, viability, motility, and morphology.^50^ While the Huangjing Zanyu Capsule, as a Chinese patent medicine (National medicine certificate, No. Z20050267), has been primarily promoted to activate blood circulation and dissipate blood stasis, studies have also indicated that this medicine can also boost sperm motility, increase sperm quantity and improve overall sperm quality.^51^, ^52^, ^53^ As such, this medicine is used for some aspects of male infertility occurring due to asthenospermia and oligospermia. We also applied this medicine to the above Chinese patients with one copy of the CCDC157 defect. This therapy was continued for 8 weeks and 1 month later, in one of them, (P1‐4) his wife then conceived, and they had a baby boy naturally. To further confirm this case as related to CCDC157, we extracted RNA from the blood samples of P1‐4 for reverse transcription experiments. The qPCR results showed that transcription levels of CCDC157 were decreased, compared to the control (Figure 8A). We then reviewed his clinic case report. Ultrasonic examination showed that his testicle size was normal, with 14.9 mL in left and 15 mL in right. Hormone tests showed that the levels of serum luteinizing hormone (LH), prolactin and follicle‐stimulating hormone (FSH), testosterone (T), estradiol (E2) and prolactin (PRL) were all within normal ranges. Semen evaluation showed that values for sperm concentration (Figure 8B), total motility (Figure 8C), progressive motility (Figure 8D) and the percentage of normal sperm (Figure 8E) were all lower than normal. However, after 3 weeks of administration with Huangjing Zanyu combined with vitamin E, the semen quality had improved, compared to that before treatment (Table S9).

Given OAT‐like characteristics in *Ccdc157* ^*−/−*^ mice and that *Ccdc157* ^*+/*−^ mice show delayed reproductive age, with significantly poorer semen quality, compared to the WT siblings, we wondered whether the Huangjing Zanyu medicine could similarly improve the semen quality of CCDC157 deficiency in mice. We carried out experiments in *Ccdc157* ^*−/−*^ and *Ccdc157* ^*+/−*^ mice where the mice were fed with the Huangjiug Zanyu from 4 to 5 weeks old until the time when their cohabited females first gave births to their offspring. Results showed that the *Ccdc157* ^*+/−*^ mice in the drug group took an average of 33 days to the first births after cohabiting with females, whereas mice in the *Ccdc157* ^*+/−*^ control group took an average of 58 days (Figure 8F). However, no offspring resulted for the *Ccdc157* ^*−/−*^ mice of either control or drug groups, even after 3 months of treatment. We analysed the sperm quality of the *Ccdc157* ^*+/−*^ mice that were treated with or without Huangjing Zanyu treatment. The motility and the percentage of normal‐headed sperms after Huangjing Zanyu treatment were much improved, compared to the control (Figure 8G–I). We checked the effect of Huangjing Zanyu medicine on the mRNA levels of *Ccdc157* by qPCR. Upregulated *Ccdc157* mRNA levels were observed in both *Ccdc157* ^*+/−*^ and *Ccdc157* ^*−/−*^ mice after treatment with the medicine (Figure S6). These data indicated that CCDC157 might have a genetic dosage effect on spermatogenesis, which can be compensated, to a certain extent, by this Chinese patent medicine of the Huangjing Zanyu.

## 3 DISCUSSION

The diagnosis and the treatment of male infertility has been a challenge due to the complex regulatory processes of spermatogenesis and with many of the molecular mechanisms that could lead to sperm failure remaining largely unelucidated. Animal models such as the mouse, zebrafish and *Drosophila* have been used to study the process of spermatogenesis and such studies have led to the discovery various gene associations with male sterility.^9^, ^10^, ^11^ However, a functionally conserved mutation shown as responsible for male infertility in models from *Drosophila* to mouse to human is an exceptionally rare finding. We identified five cases of sporadic and one pedigree of OAT carrying non‐frameshifting deletions in the *CCDC157* gene.

For *Ccdc157* ^*−/−*^ mice, in addition to dramatically reduced sperm numbers, the improper formation and detachment of acrosomes, misshapen nuclei, mis‐localization of Golgi apparatus and cytoplasmic retention in the existing sperm are all factors which would be likely to negatively influence fertilization potential, particularly compromising the ability of the acrosome for penetration through the zona pellucida of the egg.^54^ Moreover, although the flagella were of normal structure, the HTCA detachment from the nucleus in *Ccdc157* ^*−/−*^ mice would also affect the sperm motility and could lead to the sperm decapitation. In *Ccdc157* ^*+/−*^ mice, males can breed but with a delayed reproduction age and with resulting offspring often eaten by their own parents. Using the Huangjin Zanyu Chinese medicine as a treatment, the oligospermia due to heterozygous mutation of *CCDC157* in mice and humans was alleviated to a significant measure. We propose that the effect of this medicine is upon the improvement of reproduction ability manifested by alterations in sperm quality and its related metabolism. Another recent study using oligoasthenospermia model rats has also showed that Huangjin Zanyu had effects on modulating the pathways involved in taurine, purine and pyrimidine, glycerolipid and glycerophospholipid and multiple amino acid metabolisms by non‐targeted metabolomics analysis.^55^ This may provide further supportive information about the role of Huangjin Zanyu medicine in the glycolipid metabolism. However further research is needed to confirm the therapeutic effect and mechanism of the Huangjin Zanyu on infertility caused by *CCDC157* deficiency or from other causes.

Among the lipid metabolism‐related genes that have been shown to be deceased in *Ccdc157* mutant mice, OSBP has been reported to bind cholesterol through the sterol‐binding domain, interact with PI4P in the Golgi through the pleckstrin homology (PH) domain and function to carry cholesterol to the Golgi.^49^, ^56^, ^57^ In OSBP2 deficient mice, sperm differentiation was abnormal without affecting the spermatogonia proliferation and meiosis, leading to round‐shaped head, curled back head or symplast spermatozoa.^49^ We also found that the lipid metabolism‐related genes such as *Vapa, Elovl6, LPAAT* and *Lpcat3* were significantly decreased in *Ccdc157* mutant mice. Knock out LPAAT3 mice, for example, display abnormal sperm morphology.^58^ Similarly, *Elovl2* has been shown to control the level of n‐6 28:5 and 30:5 fatty acids in the testis with *Elovl2* ^−/−^ mice displaying only spermatogonia and primary spermatocytes, without mature sperm.^59^ Taken together, our findings may suggest that CCDC157 also acts as a potent regulator to control the lipid metabolism, therefore influencing sperm plasma membrane remodelling during spermatogenesis.

During acrosome biogenesis, cytosol vesicles bud from the trans‐Golgi network and bind to the acroplaxome.^60^ These proacrosomal vesicles eventually fuse to form the acrosome through a complex process including appropriate sorting, trafficking and fusion of intracellular vesicles to the correct sub‐cellular location.^17^ Various genes have been reported to be involved in this process. In these, PICK and GOPC co‐localize to trans‐Golgi vesicles. The lack of GOPC causes malformation of the acrosome.^19^ PICK1 deficiency has also been shown to disrupt acrosome formation and render male mice infertile^40^ in a manner similar to the cases of Ccdc157 deficiency shown in this paper. Hrb is required for docking and fusion of proacrosomal vesicles. Hrb mutant mice are infertile and display round‐headed spermatozoa that lack an acrosome.^41^ Acrosomal matrix genes, SPACA1, Csnk2a2 and ZPBP1 have also been seen to affect the structure of the acroplaxome.^45^ Defects in these result in the detachment of the acrosome from nucleus,^43^ acrosomal fragmentation or disassembly of the protein matrix,^44^ respectively. In *Ccdc157‐d*eficient germ cells, we detected significant decreases in all the above genes. An abnormal Golgi membrane morphology was observed in *Ccdc157*‐depleted mouse cells which also corresponded to results of studies using human Hela cells.^38^ These observations suggest that CCDC157 may play an important role during sperm acrosome formation through maintaining the structure and function of Golgi membranes.

In conclusion, we have identified a male infertile‐associated gene, CCDC157, as indispensable for spermiogenesis. Its deficiency may result in patients with an OAT diagnosis. By analysing *Ccdc157*‐KO mice, we demonstrated that mammalian CCDC157, as for *Drosophila Pif1A*, has key functions in regulating Golgi apparatus and acrosome formation during spermiogenesis (Figure 8J). Related to the clinical applications of this study, we not only found a family with *CCDC157* defects in the APC_basic domain, but also found a heterozygosis case of CCDC157 with defect in SMC_N super family domain. The Chinese medicine, Huangjin Zanyu, has a potential for the improvement of fertility ability in both patients and mice carrying the heterozygous mutation of CCDC157. Our study reveals that CCDC157 might act as a molecular target for diagnosis of OAT.

## 4 MATERIALS AND METHODS

### 4.1 Ethics statement

All animal studies were conducted in compliance with the Zhejiang University Experimental Animal Welfare Ethics Review Committee (file no. ZJU20210154). All human studies have been approved by the Ethical Committee of the Women's Hospital, Zhejiang University School of Medicine (file no. IRB‐20210187‐R) and the institutional human ethics committee at the University of Science and Technology of China (USTC) (with the approval number—file no. USTCEC20190148). All participants signed a document of informed consent before participating in the study.

### 4.2 WES and variant screening

Total genomic DNA was isolated from the peripheral blood of study samples. Whole‐exome capture and sequencing were performed using the AlExome Enrichment Kit V1 (iGeneTech, Beijing, China) and Hiseq2000 platform (Illumina, San Diego, CA, USA) following the standard procedures. The reads were aligned to the human genome reference assembly (hg19) using Burrows‐Wheeler Aligner (BWA) with default parameters. Then the Picard software (http://picard.sourceforge.net/) was employed to remove polymerase chain reaction (PCR) duplicates. DNA sequence variants were called using Genome Analysis Toolkit HaplotypeCaller (http://www.broadinstitute.org/gatk/).Variants were filtered as we previously described [J Exp Med. 2020 Feb 3;217(2):e20182365., Genet Med. 2019 Jan;21(1):62‐70.] with some minor modifications. In brief, considering that the individuals were born to a consanguineous marriage, variants following recessive inheritance pattern in the sequenced family members were kept for further screening. Variants meeting the following conditions were given preference: (1) MAF <0.01 in the 1000 Genomes, ESP6500, ExAC and Genome Aggregation Database; (2) loss‐of‐function variants or potentially deleterious missense variants as predicted by seven software packages including Sorting Intolerant From Tolerant (SIFT), PolyPhen‐2, MutationTaster and others; and (3) variants carried by genes expressed in testis.

### 4.3 Animals

The mice we used were C57BL/6. The *Ccdc157* knockout allele was generated using CRISPR/Cas9 from Cyagen Biosciences Inc (China). The two gRNA were gRNA1 (matching reverse strand of gene): CTCTGAGAGCGGCCTATGGTGGG. gRNA2 (matching reverse strand of gene): GGGAGGATCCATCCAACCTAGGG. For genotyping of Ccdc157, genomic DNA from the tail of newborn mice was extracted, and PCR was carried out by the following primers, P1‐F: 5′‐GCTCTGCCTCCTTCTGAGTTAG‐3′; P1‐R:5′‐GTTTGTCTTCTGACCACACTCC‐3′; P2‐F: 5′‐CTCGTCTCAATAAACATGTGGG‐3′; P2‐R:5′‐CCACCTTTGTCTTTAGGTCACA‐3′. All mice were maintained in the standard specific pathogen‐free facility of the Animal Laboratory Center of Zhejiang University at a temperature at 22–24°Cand a 12 h dark/light cycle (light on from 7 a.m. to 7 p.m.). Four to five mice were kept per cage and could freely access to food and water ad libitum. The mice were sacrificed by cervical dislocation.

### 4.4 Animal grouping and drug administration

After genotyping the mice, different genotypic males were set up in different groups namely the C57BL/6 control group, the *Ccdc157* ^*+/−*^ group, and the *Ccdc157* ^*−/−*^group. In each group, 10 four‐ to five‐week‐old mice were randomly divided into two subgroups, the gavage feeding with water subgroup and the gavage feeding with Huangjing Zanyu subgroup.

We dissolved the powder of the Huangjing Zanyu capsule (National medicine certificate, No. Z20050267 from Shanghai Xinya HanJiang Co., Ltd) in distilled water and mixed it to a uniform 0.37 g/mL, heating the solution to 37°C before administration. The Huangjing Zanyu groups were given 0.4 mL 0.37 g/mL Huangjing Zanyu and the water groups were given 0.4 mL water once a day until their cohabited female mice gave births to their first offspring. The male mice were then tested for sperm quality. For the *Ccdc157* ^*−/−*^ groups, the female mice had still failed to display pregnancy after 3 months treatment with Huangjing Zanyu.

### 4.5 Western blot analysis

To detect CCDC157 protein, fresh dissected testes were homogenized in a lysis buffer (1xRIPA buffer: 50 mM Tris–HCl, pH 8.0, 150 mM NaCl, 1% IGEPAL CA‐630, 0.5% sodium deoxycholate, 0.1% SDS) and protease inhibitor cocktail (Roche). The lysates were cleared by centrifugation at 14,000 xg for 10 min at 4°C. Equivalent amounts of total protein per sample were diluted with loading buffer, boiled for 5 min and stored at −20°C until use. Samples were subjected to sodium dodecyl sulphate polyacrylamide gel electrophoresis (SDS/PAGE) and transferred to a PVDF membrane and immunoblotted at 4°C overnight with antibodies. The primary antibodies used were rabbit anti‐CCDC157 (1:1000, Genetex, GTX45090), rabbit anti‐CCDC157 (1:1000, Abcam, ab243450), rabbit anti‐GOPC (1:1000, proteintech, 12163‐1‐AP), rabbit anti‐HRB (1:1000, Affinity, DF2639), rabbit anti‐GAPDH (1:10,000, proteintech, 10494‐1‐AP) and mouse anti‐tubulin (1:1000). The secondary antibodies were HRP‐conjugated (1:5000). Blots were treated with the ChemiLucent ECL detection reagents (Millipore) and protein bands were visualized using the Chemiluminescence Imaging System (Clinx Science Instruments, Shanghai, China).

### 4.6 Histology on tissue sections

The testes and epididymides of adult mice were fixed in 4% formaldehyde solution in PBS for at least 24 h, paraffin embedded, and 5 μm sections were stained with haematoxylin and eosin, using standard procedures. Then sections were examined using a Nikon 80i microscope (Nikon, Tokyo, Japan).

### 4.7 Transmission electron microscopy

Testicular tissues were fixed in 2.5% glutaraldehyde at 4°C overnight. After several rinses in PBS, post‐fixation was performed for 1.5 h at room temperature in 1% osmium tetroxide. After three rinses in PBS, samples were dehydrated in a graded series of ethanol (30%, 50%, 75%, 80%, 90%, 95% and 100%), and embedded in epoxy resin through increasing concentrations (50%, 75% and 100%), then cured at 70°C overnight. Electron micrographs were taken on a Hitachi H‐7650 TEM.

### 4.8 Immunofluorescence

Male mice were sacrificed and the tissues were immediately embedded in optimum cutting temperature compound (OCT, Tissue‐Tek) and cut into 8 μm sections using a microtome‐cryostat (LeicaCM1950, Wetzlar, Germany). Frozen sections were fixed with 4% paraformaldehyde for 20 min and washed in PBS three times. After being blocked with 5% BSA for 30 min, the sections were incubated with primary antibody (sp56, QED Bioscience, 55101; Lectin PNA, Alexa Fluor™ 488 Conjugate, Thermo Fisher Scientific, L21409; GM130, BD Bioscience, 610822; TGN46, Abcam, ab16059) at 4°C overnight. After being washed with PBS, the samples were incubated with secondary antibody, diluted with PBS for 1 h at 37°C, washed with PBS and stained with DAPI. The slides were then mounted and viewed. The images were acquired using an FV1000 confocal laser scanning microscope (Olympus, Tokyo, Japan).

### 4.9 Cell cultures and siRNA‐mediated knockdown

The GC‐2 cell line^61^ was grown in incomplete medium consisting of DMEM containing 10% FBS (Gibco), 100 U/mL penicillin and 100 μg/mL streptomycin (Sigma‐Aldrich) at 37°C with 5% CO_2_.

Specific small interfering RNA (siRNA) was transfected in GC‐2 cells using EndoFectin Maxtransfection reagent (Gene Copoeia; EF014) following the manufacturer's recommendations. siDirect (http://sidirect2.rnai.jp/). The corresponding web‐based online software was used to design target‐specific dsRNA to avoid off‐target silencing effects. The siRNA sequences used were as follows: Negative control: 5′‐UUCUCCGAACGUGUCACGUTT‐3′, 5′‐ACGUGACACGUUCGGAGAATT‐3′; Ccdc157: 5′‐GAAACAUGUGACCUAAAGACATT‐3′, 5′‐UGUCUUUAGGUCACAUGUUUCTT‐3′.

### 4.10 Assessment of mouse sperm quality

Epididymides from male mice were dissected and cut open with fine scissors. For spermatozoa counting, spermatozoa were collected and fixed in 2% formaldehyde for 10 min at 25°C. After washing and resuspending in PBS buffer, the spermatozoa were counted using a haemocytometer chamber under an optical microscope. The sperm count was calculated as described.^62^ For spermatozoa morphology assessment, sperm were viewed randomly under a 200× magnification to determine the percentage of sperm with abnormal head shapes (non‐sickle shaped). At least 200 sperms in five or more fields of view were evaluated for each experimental condition. For the spermatozoa motility assay, assessment was carried out following the previously described procedure.^63^ Briefly, the sperm from the epididymis was squeezed and placed in warm PBS. Ten minutes after collection of sperm, sperm motility was observed on a pre‐warmed slide using a ZEIS Saxio observer 5 inverted microscope. Eight fields were analysed for each sperm sample. Individual spermatozoa were tracked using Image J (NIH, Bethesda, MD) and the plug‐in MTrack J. Sperm motility was calculated according to the curve velocity (VCL), which is the curvilinear distance (DCL) of spermatozoa counted in seconds (VCL = DCL/*t*).^64^

### 4.11 Quantitative RT‐PCR

Total RNA was extracted using Trizol reagent (Thermo Fisher Scientific). cDNA was synthesized from the RNA samples with a First‐Strand cDNA Synthesis Kit (Thermo Fisher Scientific). We used the Power SYBR Green PCR Master Mix (Thermo Fisher Scientific) to conduct RT‐PCRs with beta‐tubulin used as a control. Real‐time PCR was performed on an ABI7900HT Fast Real‐Time PCR machine (Thermo Fisher Scientific). The PCR primers used are provided in Table S10.

### 4.12 Statistical analysis

Each of the experiments was performed at least three times with data expressed as the mean ± SD. Significant difference was evaluated using a Student's *t*‐test in Microsoft Excel.

## AUTHOR CONTRIBUTIONS

**Huimei Zheng:** Data curation (equal); funding acquisition (equal); investigation (equal); software (equal); writing – original draft (equal). **Chenjia Gong:** Data curation (equal); methodology (equal); resources (equal); writing – review and editing (equal). **Jingping Li:** Data curation (equal); investigation (equal); resources (equal). **Jiaru Hou:** Investigation (equal); methodology (equal). **Xinhan Gong:** Investigation (equal); methodology (equal). **Xinhai Zhu:** Investigation (equal); methodology (equal); software (equal). **Huan Deng:** Investigation (equal); validation (equal). **Haoyue Wu:** Investigation (equal). **Fengbin Zhang:** Resources (equal). **Qinghua Shi:** Resources (equal); writing – review and editing (equal). **Jianteng Zhou:** Methodology (equal); resources (equal). **Baolu Shi:** Methodology (equal); resources (equal). **Xiaohang Yang:** Conceptualization (equal); formal analysis (equal); project administration (equal); supervision (equal); writing – review and editing (equal). **Yongmei Xi:** Conceptualization (equal); funding acquisition (equal); supervision (equal); writing – review and editing (equal).

## FUNDING INFORMATION

This study was supported by grants from the Fundamental Research Funds for the Central Universities (17221012101), National Key R&D Program of China (2018YFC1004900) and the National Natural Science Foundation of China (31801230).

## CONFLICT OF INTEREST STATEMENT

The authors declare that they have no competing interests.

## Supporting information


Data S1.

Table S3.

Table S4.

Table S5.

Table S6.

Table S7.

Table S8.

## Data Availability

The data are available from the corresponding author upon reasonable request.
