# Supplementary material for: CCDC157 is essential for sperm differentiation and shows oligoasthenoteratozoospermia‐related mutations in men
Source: J Cell Mol Med. 2024 Mar 20;28(7):e18215. doi: 10.1111/jcmm.18215 (PMC10955179; doi:10.1111/jcmm.18215)
Supplement: Supplementary file 1 — Data S1. [file JCMM-28-e18215-s001.docx]

# Supplement Figure 1


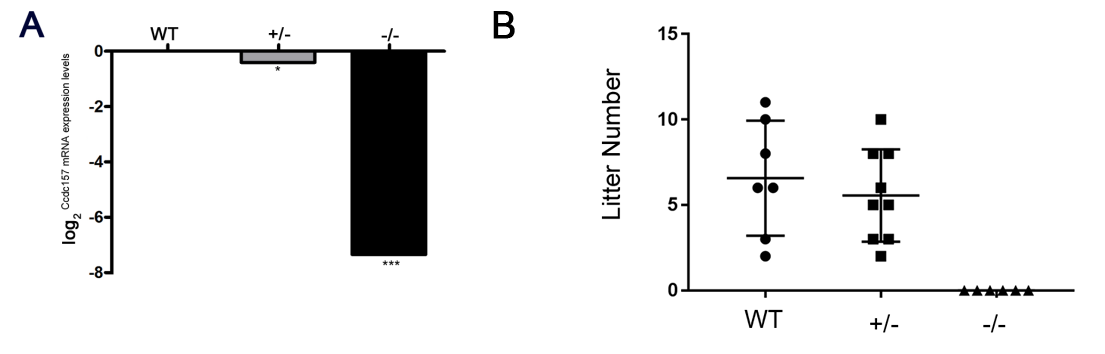


(A) Real-time PCR analysis of *Ccdc157* transcriptional levels of wild type animals, *Ccdc157^+/-^* and *Ccdc157^-/-^* mice. (B) Number of live pups per litter resulting from crosses between 8-week- old males and age-matched, WT females.

# Supplement Figure 2


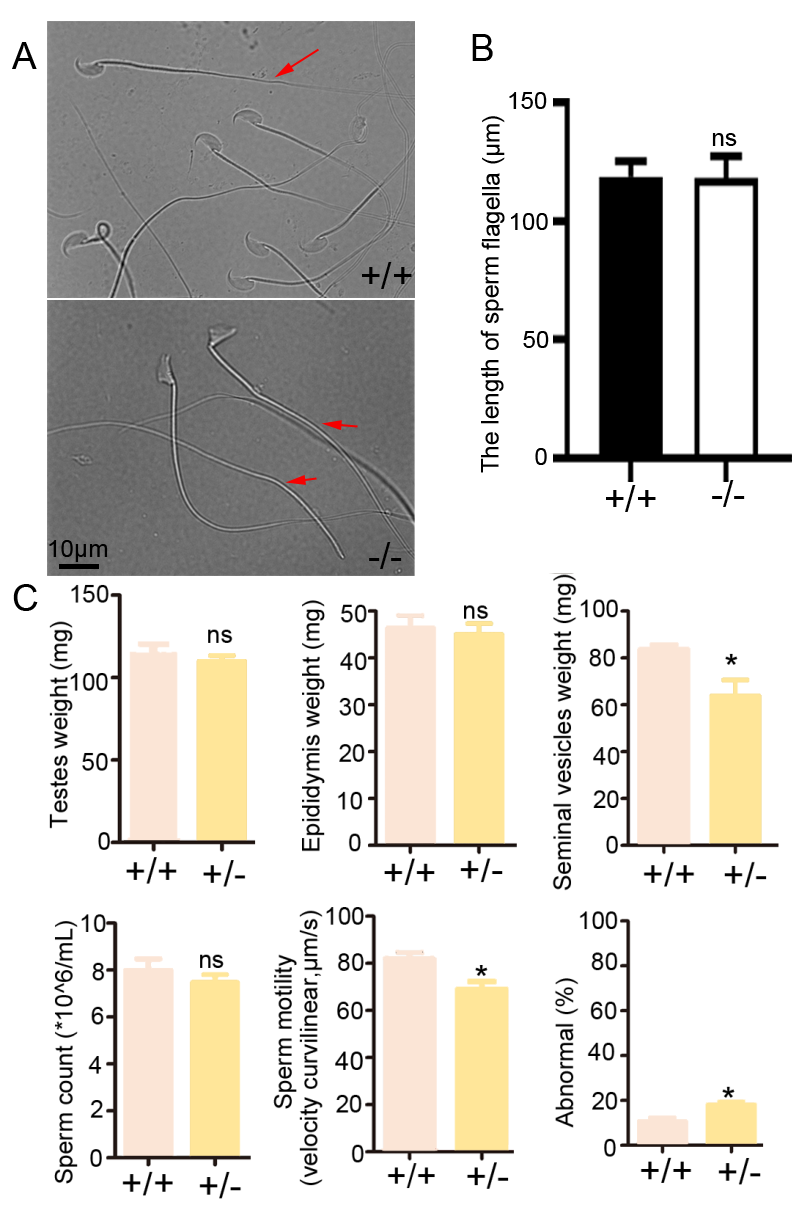


(A) Microscopic images of epididymal sperm of C57BL/6 and *Ccdc157^-/-^*; red arrows indicated the sperm flagella. (B) The length of sperm flagella between wild-type and *Ccdc157^-/-^* mice. (C) Quantitative analysis of testis weight, epididymis weight, seminal vesicle weight, concentrations of epididymal sperm, sperm motility and the percentage of epididymal sperm showing abnormal-heads from 8-week-old C57BL/6 and *Ccdc157^+/-^*.

# Supplement Figure 3

**
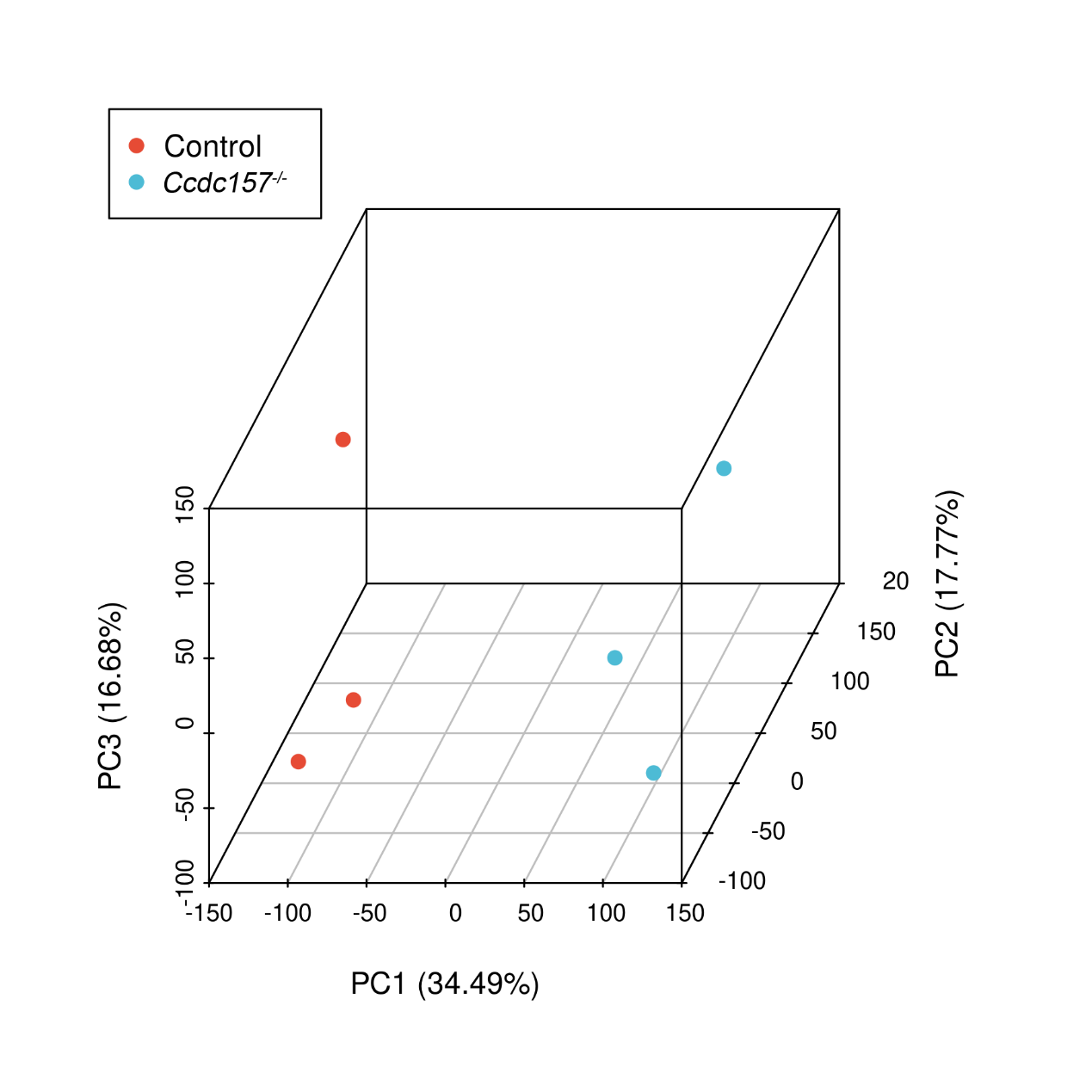
**

PCA plot showing clustered samples based on the *Ccdc157* mutant and WT genotype. Each point represents replicate sample for each genotype.

# Supplement Figure 4

(A) Western Blot analysis of the testes extracts from Ccdc157^+/+^ and Ccdc157^-/-^. Lysates were probed with an anti-COPC antibody and anti- GAPDH antibody. (A’) Western Blot analysis of the testes extracts from Ccdc157^+/+^ and Ccdc157^-/-^. Lysates were probed with an anti-GAPDH antibody and anti- HRB antibody.

# Supplement Figure 5


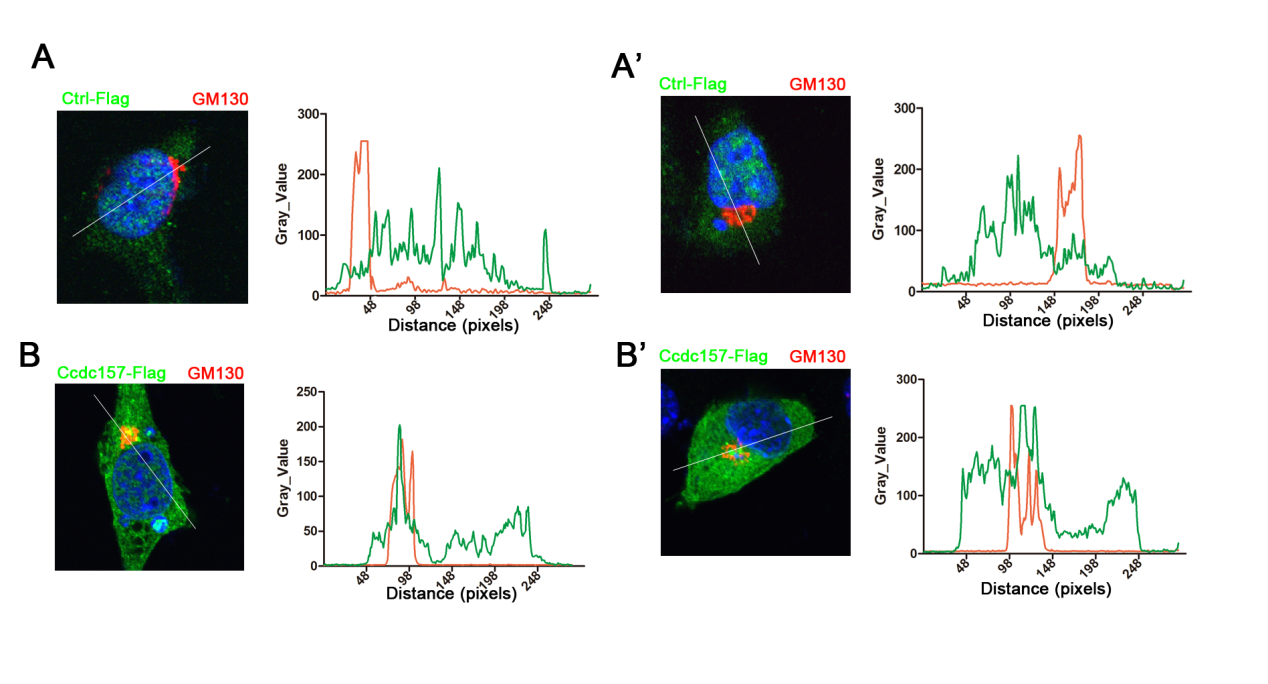


**Analyzing on the co-localization of Ccdc157 and GM130 in a GC-2 cell line.** (A , A’) Right panel: GC-2 cells were transfected with indicated pcDNA3.1-flag plasmid and after 2 days, analyzed by immunofluorescence microscopy using an anti-GM130 antibody. Left panel: Intensity profile graphs of GM130 co-localization derived from confocal micrographs shown on the right panel.

(B, B’) Right panel: GC-2 cells were transfected with indicated pcDNA3.1-Ccdc157-flag plasmid and after 2 days, analyzed by immunofluorescence microscopy using an anti-GM130 antibody. Left panel: Intensity profile graphs of GM130 co-localization derived from confocal micrographs shown on the right panel.

# Supplement Figure 6


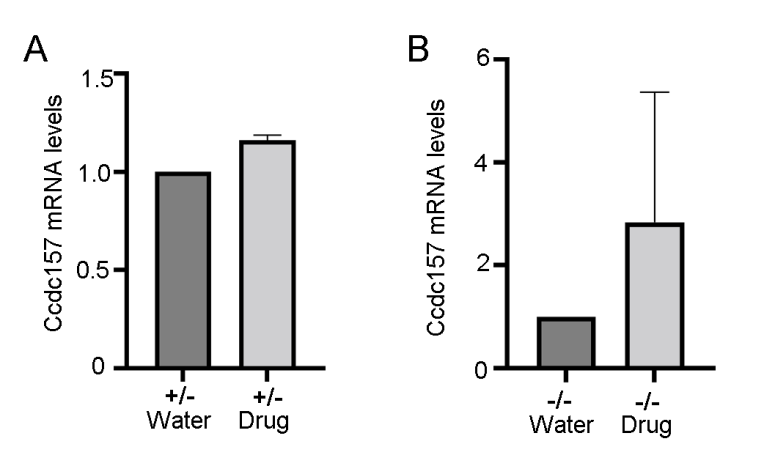


(A) Real-time PCR analysis of *Ccdc157* transcriptional levels of *Ccdc157^+/-^* animals treated with Huangjing Zanyu Capsule or water. (B) Real-time PCR analysis of *Ccdc157* transcriptional levels of *Ccdc157^-/-^* animals treated with Huangjing Zanyu Capsule or water.

# Supplemental Table 1.

**Clinical characteristics of the patient IV:6**

| Subject | IV:6  (PK.INF-672) | Reference values^d^ |
| --- | --- | --- |
| **Basic Information** | | |
| Reproductive status | Infertility |  |
| Age(y)^a^ | 38 |  |
| BMI | 26.7 |  |
| Age(y) of marriage^b^ | 18 |  |
| **Semen parameters** | | |
| Semen volume(ml) | 2.5±0.5 | >1.5 |
| Sperm concentration(nx10/ml) | 5±5 | >1.5*10^6^ |
| Total motility(%) | 0 | >40 |
| No motility (%) | 100 |  |
| Normal morphology (%) | 60 | >4 |
| Abnormal morphology (%) | 40 |  |
| **Physical Examination** | | |
| External genitalia | Normal |  |
| Secondary traits | Normal |  |

^a^ The current age.

^b^ The year of marriage.

^c^ Semen analysis were done by two independent experiments. Data are presented as mean ± SEM.

^d^ Reference standards were published in WHO(2010).

# Supplemental Table 2.

**The average days that female mice give the first births after cohabiting**

|  | +/+ female | -/- female | +/- female |
| --- | --- | --- | --- |
| +/+ male | 28 | 36 | 33 |
| -/- male | Females were not conceived | Females were not conceived | Females were not conceived |
| +/-male | We didn't do this | 58 | 56 |

# Supplemental Table 9.

**The semen quality of the patient P1-4 before (-) and after (+) the medical treatment.**

| Drugs | Sperm  concentrations | Total motility | Progressive motility | Normal shaped  sperm |
| --- | --- | --- | --- | --- |
| - | 3*10^6^/ml | 18% | 12% | 2% |
| + | 7.6*10/ml | 49.9% | 37% | 2% |

# Supplemental Table 10.

Primers for RT-PCR

| m-osbp2-qf | CTATGATCAACGCCTGTAGG |
| --- | --- |
| m-osbp2-qr | CCGTTCAAGACTGTTGTGCT |
| m-vapa-qf | TTCAAAGGCCCCTTCACAG |
| m-vapa-qr | GTTGCAGCATTACTGAAACAGTC |
| m-lpcat3-qf | TCTTCCTGGGCTACCCGT |
| m-lpcat3-qr | TGAGTCGCAGGATGAGGAA |
| m-mboat2-qf | GCATGCAGCAATGTTGCTTTGT |
| m-mboat2-qr | CCGAAACATCCCGTCGTGAA |
| m-elovl6-qf | TATTCGGTGCTCTTCGAACTG |
| m-elovl6-qr | GGTGCTTTGCTGAGCACAA |
| m-Pick-qf | TAGGCGAGCCGCTATACCGAG |
| m-Pick-qr | ATGTCCTGGACGTGCTTCTG |
| m-Gopc-qf | CTGCTTGTCAGACTCGTGGAG |
| m-Gopc-qr | CTGAGGTTCCCGCCTTTCT |
| m-Spaca-qf | GAGGTGCGAACAAGACAAGG |
| m-Spaca-qr | AGAAAGCCGGACGTTACAGG |
| m-Ccdc136-qf | ACTCTGCCACTGAACATGAGA |
| m-Ccdc136-qr | AAGTCGGAGATGCTTAGATTAGAT |
| m-Zpbp-qf | TTGGACATTTGGCTCGACTGCC |
| m-Zpbp-qr | CCTTTTGGCCCATGCCATTGG |
| m-Csnk2a2-qf | ACATTCACGGAAGCGCTGGG |
| m-Csnk2a2-qr | ACGGTGTTCTCAGCACAAGGC |
| m-Hrb-qf | AGTTTCCAGCAGCCTGCCTTC |
| m-Hrb-qr | GAGCTTCCTGTTGGAAGTTGTCCT |
| m-Spata16-qf | CTTTGCAGCAAAGGAGCAGCTC |
| m-Spata16-qr | CGGCTGCCTGACGAAGATGG |
| m-b-actin-qf | AAGTGTGACGTTGACATCCG |
| m-b-actin-qr | GATCCACATCTGCTGGAAGG |
| mCCDC157-1-qF | CAGAGCATCCCTGTCAGAGT |
| mCCDC157-1-qR | ATGTTTATTGAGACGAGTCAGGT |
| h-ccdc157-q-f1 | TCAGTAAGCATGTGGAGGC |
| h-ccdc157-q-r1 | TCACTTGTTTCTGTGAGCAG |
| h-ccdc157-q-f2 | CCACCAGGAGTCTCTGCAG |
| h-ccdc157-q-r2 | CAGGTCCGTAGTCGCCTG |
| h- GAPDH -qf | ACCACCATGGAGAAGGCTGG |
| h- GAPDH -qr | CTCAGTGTAGCCCAGGATGC |

Table S3--The differentially expressed genes between Ccdc157 mutant and control

Table S4-- Up-regulated gene-enriched GO terms in Ccdc157 mutant compared with control

Table S5-- Down-regulated gene-enriched GO terms in Ccdc157 mutant compared with control

Table S6--The expression levels of Golgi related genes between Ccdc157 mutant and control

Table S7—Up-regulated gene –enriched KEGG analysis in Ccdc157 mutant compared with control

Table S8-- Down-regulated gene –enriched KEGG analysis in Ccdc157 mutant compared with control
